# Postpartum depression: a developed and validated model predicting individual risk in new mothers

**DOI:** 10.1038/s41398-022-02190-8

**Published:** 2022-09-30

**Authors:** Trine Munk-Olsen, Xiaoqin Liu, Kathrine Bang Madsen, Mette-Marie Zacher Kjeldsen, Liselotte Vogdrup Petersen, Veerle Bergink, Alkistis Skalkidou, Simone N. Vigod, Vibe G. Frokjaer, Carsten B. Pedersen, Merete L. Maegbaek

**Affiliations:** 1 Department of Clinical Research, University of Southern Denmark, Odense, Denmark; 2 National Centre for Register-Based Research, Aarhus BSS, Aarhus University, Aarhus, Denmark; 3 Department of Psychiatry, Erasmus Medical Centre Rotterdam, Rotterdam, The Netherlands; 4 Department of Psychiatry, Icahn School of Medicine at Mount Sinai, New York City, NY USA; 5 Department of Women’s and Children’s Health, Uppsala University, Uppsala, Sweden; 6 Women’s College Hospital and Women’s College Research Institute, Department of Psychiatry, University of Toronto, Toronto, ON Canada; 7 Mental Health Services, Capital Region of Denmark, Copenhagen, Denmark; 8 Neurobiology Research Unit, Copenhagen University Hospital, Copenhagen, Denmark; 9 Department of Clinical Medicine, Faculty of Health and Medical Sciences, Copenhagen University, Copenhagen, Denmark; 10 Centre for Integrated Register-based Research, CIRRAU, Aarhus University, Aarhus, Denmark

**Keywords:** Depression, Scientific community

## Abstract

Postpartum depression (PPD) is a serious condition associated with potentially tragic outcomes, and in an ideal world PPDs should be prevented. Risk prediction models have been developed in psychiatry estimating an individual’s probability of developing a specific condition, and recently a few models have also emerged within the field of PPD research, although none are implemented in clinical care. For the present study we aimed to develop and validate a prediction model to assess individualized risk of PPD and provide a tentative template for individualized risk calculation offering opportunities for additional external validation of this tool. Danish population registers served as our data sources and PPD was defined as recorded contact to a psychiatric treatment facility (ICD-10 code DF32-33) or redeemed antidepressant prescriptions (ATC code N06A), resulting in a sample of 6,402 PPD cases (development sample) and 2,379 (validation sample). Candidate predictors covered background information including cohabitating status, age, education, and previous psychiatric episodes in index mother (Core model), additional variables related to pregnancy and childbirth (Extended model), and further health information about the mother and her family (Extended+ model). Results indicated our recalibrated Extended model with 14 variables achieved highest performance with satisfying calibration and discrimination. Previous psychiatric history, maternal age, low education, and hyperemesis gravidarum were the most important predictors. Moving forward, external validation of the model represents the next step, while considering who will benefit from preventive PPD interventions, as well as considering potential consequences from false positive and negative test results, defined through different threshold values.

## Introduction

Postpartum depression (PPD) is a serious condition with documented negative and potentially tragic consequences, including recurrence, self-harm, and suicide [1–3]. Prevalence of PPD is around 13%, but ranges substantially depending on case definition criteria and study population [4–7], and risk factors, among others, including past history of depression and pregnancy/obstetric complications [4, 8–10].

In an ideal world, PPD should be prevented, and interventions to do this have been developed and tested [11]. For targeted interventions, any effort to successfully identify individual women at particularly high risk of PPD is consequently preferable and also cost-effective [12]. Unfortunately, no such tools exist that are sufficiently validated [12], which directly impedes and averts the initiation of early treatment and individualized risk management in clinical care. So far, clinical practice can only apply a pragmatic approach based on a Grade B recommendation: Provide counseling interventions to women with one or more established risk factors, including a history of depressive episodes, current depressive symptoms, low socioeconomic status, recent intimate partner violence, or a history of significant negative life events [12]. However, such an approach will (A) provide counseling to women who despite having identified risk factors do not develop PPD and (B) miss the opportunity to help a group of women who will develop PPD without having any of the outlined risk factors. Consequently, this pragmatic approach will capture some high-risk PPD individuals but is at its best imprecise.

Risk prediction models have been developed in psychiatry in recent years, aiming to estimate an individual’s probability of a selected condition, including diagnostic, prognostic, or predictive models in response to interventions [13]. Examples of these tools include models to predict readmission [14], and disease-specific risks for, e.g., psychotic or affective disorders, and posttraumatic stress [13, 15], and mainly recently, models aimed at predicting PPD [16–21]. In comparison, there are several examples of risk prediction models outside the field of psychiatry, which are implemented in daily clinical practice. These include, for example, the identification of persons at high risk of breast cancer and cardiovascular disease [22–24]. A common denominator for these models is that the tools are dynamic and have been developed, fine-tuned, and trained across a longer period and have taken advantage of input from validation in external datasets and expansion of the predictor variables [25–28].

For the present study, we aimed to develop and validate a prediction model to assess an individualized risk of PPD, and furthermore provide a tentative template for individualized risk calculation, offering opportunities for additional external validation of this tool.

## Methods

### Data sources

Danish population registers served as our data sources, and linkage was possible as all individuals alive and living in Denmark from 1968 and onwards are assigned a unique identification number registered in the Danish Civil Registration System (CRS) [29]. This identification number enables linkage within and between registers and provides information on vital status and family relations. The Danish National Patient Registry contains information on all admitted patients with somatic diseases from 1977, and the Danish Psychiatric Central Research Registry (PCR) holds data on all patients admitted to psychiatric hospitals from 1969 [30]. Both registries also contain data on outpatient and emergency visits from January 1, 1995. The Danish National Prescription Registry (NPR) provides data on all redeemed prescriptions from 1995 [31], and contains the anatomical therapeutic chemical (ATC) classification codes and the dispensing date. The Danish Medical Birth Registry (MFR) includes data on all live births and stillbirths and contains information on gestational age and birth complications from 1973 [32]. Furthermore, data on socioeconomic status (education and cohabiting information) was obtained from the Population Statistics Register and the Danish Student Register and Qualification Register [33].

### Study design and population

Through CRS, we identified Danish women who gave birth to their first live-born singleton.

**Fig. 1:**
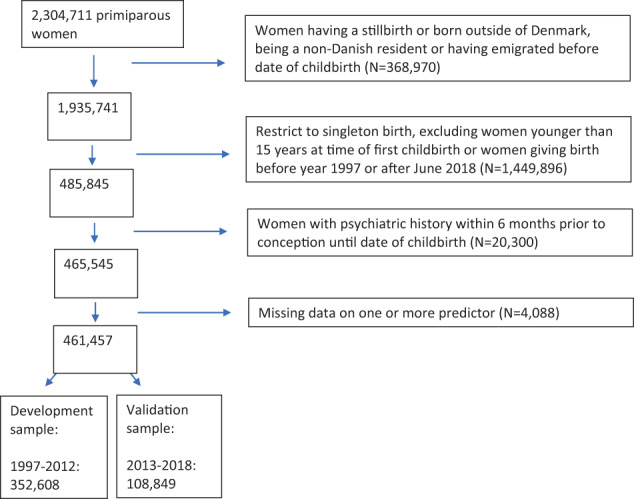
Study population details.

To ensure sufficient registry information prior to and after childbirth, we only included Danish-born women aged 15 years or older and who resided in Denmark at delivery between January 1, 1997, and June 30, 2018 (*N* = 485,845). To ensure PPD was an incident psychiatric episode, we excluded 20,300 women with a psychiatric history within 6 months prior to conception until date of childbirth (International Classification of Diseases, 10th Revision (ICD-10) codes: F00-99 and ATC-codes: N03-N07). Furthermore, 4,088 had missing information on at least one predictor variable, leaving 461,457 women for the analysis: 352,608 (development sample) and 108,849 (validation sample) (Fig. 1).

### Definition of PPD

PPD was our outcome of interest and was defined as either a recorded contact to a specialized psychiatric treatment facility as in- or outpatient visit (ICD-10: codes: DF32-33) or as a redeemed antidepressant prescription in primary care (ATC code N06A) within 6 months after childbirth.

### Identification and definition of candidate predictors

To develop our prediction model to assess the risk of PPD, we selected a set of predictors previously shown to be associated with PPD, which are easy to identify and readily available in clinical screening and care. We defined three prediction models with varying numbers of included candidate predictors: (1) Core model, (2) Extended model, and (3) Extended+ model. See details regarding identification and definition of all predictor variables in Supplementary, Table 1.

### Core model—predictor variables including background information

In the Core model, we included predictor variables covering identified PPD risk factors related to background information, including cohabitation status (married or cohabiting/single, divorced, widowed), age, education, and previous psychiatric episodes of the index mother.

### Extended model—adding PPD risk factors before and during pregnancy and around childbirth

In the Extended model, we included the predictors from the Core model and added identified risk factors from previous work from our group, including the following dichotomous (yes/no) variables registered during pregnancy: Hyperemesis gravidarum, eclampsia, preeclampsia, gestational hypertension, and gestational diabetes. Predictor variables around childbirth included postpartum hemorrhage, preterm birth, and acute cesarean section (C-section), while predictor variables before pregnancy included history of previous stillbirths and spontaneous or induced abortions [8].

### Extended+ model—adding detailed information on health of the index mother and her family

For the Extended+ model, we added additional PPD risk factor variables to the Extended model. The added information constituted data that not all women will know and be able to disclose, including psychiatric history in at least one parent before date of childbirth and somatic comorbidities defined by Charlson Comorbidity index [34].

### Statistical analyses

#### Model development

Complete case analysis was done as only a few women (*N* = 4088, 0.9%) had missing values on either education and/or cohabitation (Fig. 1) [35]. Applying a non-random split [36], the remaining 461,457 mothers were divided into a development and a validation sample based on the calendar year of childbirth: women with a first-time birth in 1997–2012 (development sample, *N* = 352,608), and women with a first-time birth in 2013–2018 (validation sample, *N* = 108,849). Note, the non-random split was chosen, instead of a random split, because it has superior properties for evaluating model performance allowing for non-random variation between the two datasets. The incidence of PPD within each of the two samples was 0.02 and the EPV (events per variable) was above 100.

We estimated the probability of PPD (yes/no) within 6 months after birth, using a logistic regression model. Within our follow-up period, 139 women either died or emigrated without a PPD diagnosis and were defined as non-PPD cases. Within the development sample, we considered the association between each predictor and the probability of PPD measured by odds ratios (ORs). The functional form used for age was assessed, examining piecewise linear functions, spline-, logarithmic- and power transformations. We used a full model approach [26], combining all the above-mentioned predictors in a multivariate logistic regression model for the three outlined models: Basic, Extended, and Extended+. We determined the best fit for each variable and found the optimal model by evaluating *R*^2^ and the Akaike information criterion (AIC), ending up with an optimally defined model for each of our three prediction models (Core, Extended, and Extended+).

#### Prediction model validation

To examine the extent to which our models can be generalized and used outside the development sample, reproducibility and transportability were assessed [26, 37]. Reproducibility measures the model performance within a similar dataset from the same population, whereas transportability measures the performance in samples that are different from the development sample but still from the same population and context. Overall, a good model performance captures both reproducibility and transportability.

#### Internal validation

Reproducibility was examined within each of the three models, considering the performance of the fitted model evaluated on the same data as it was developed (internal validation). A fitted model typically over-performs because it is evaluated within the same data, and this ‘optimism’ in performance is a measure of reproducibility. Constructing 200 bootstrapped samples at random with replacement using the function “validate” in R package rms, we estimated a logistic regression model in each sample and evaluated each of these with the original sample. The differences in the regression slope in all 200 samples were subsequently combined and pooled, as a measure of average optimism and used to shrink the original model to the mean, improving stability to enhance reproducibility in new datasets [38].

#### Internal-external validation

Transportability was assessed using temporal validation to evaluate model performance while considering the two different time periods for development and validation [39]. The three models were developed within women with a first-time birth in 1997–2012 (development sample), and each of the developed models was evaluated within more recent mothers (validation sample), predicting the risk of PPD in women with a first-time birth in 2013–2018 (internal-external validation [39]). If the predicted risk of PPD was far from the actual risk, we adjusted the models accordingly using recalibration techniques [40], as explained below.

#### Model performance assessment

The performance of the models was assessed by discrimination and calibration. Discrimination describes the models’ ability to distinguish between women with and without the event. It is measured by the c-statistic corresponding to the area under the ROC curve, representing the probability that within a randomly selected pair of women (one with and one without PPD), the woman with PPD was assigned a higher predicted probability than the woman without PPD.

Calibration measures if the model is precise in predicting the observed probability [41]. We considered this by plotting the predicted probabilities versus observed probabilities, where the identity line represents a perfect calibration. The predicted probabilities were explained by the logit model with the linear predictor LP_*i*_ = *α*_*dev*_ + *β*_dev_*X_*i*_, where (α_dev_, β_dev_) is the coefficients estimated within the development sample and *i* indicates which dataset the predictors belong to. As we consider logistic regression with a dichotomous outcome, the observed probabilities were smoothed by the loess algorithm. The calibration slope, *b*_overall_, was estimated from the recalibration model, logit(Y_*val*_ = 1) *= a* + *b*_overall_ * LP_val_, and indicates whether the model is over- or underfitting depending on whether *b*_overall_ is below or above 1, respectively. When the slope equals 1, calibration-in-the-large is the calibration intercept, *a*, which describes whether the model overestimates or underestimates the probability of PPD depending on whether it is below or above 0, respectively. The calibration slope and intercept were used to recalibrate the model to gain better calibration [40]. Additionally, we considered the le Cessie-van Houwelingen-Copas-Hosmer (CHCH) unweighted sum of squares test for global goodness of fit [42].

The final model is presented in a nomogram, illustrating how much each predictor affects the probability of PPD. Furthermore, a risk calculator is available online to calculate the probability of PPD based on the combination of an individual woman’s covariates (https://ncrr-au.shinyapps.io/PPDRiskCalc/). Note, the provided risk calculator is at present not ready for implementation into clinical care and is provided solely for validation purposes. TRIPOD guidelines were followed for the development and validation of our prediction model [36]. All analyses were performed using the R software [43] version 4.1.1, using the following packages: rms [44], DescTools [45], pROC [46], Hmisc [47], and caret [48], and shiny [49].

#### Ethics

Using population register data for the present study was approved by the Danish Data Protection Agency. No informed consent is required for these types of studies in accordance with Danish legislation.

## Results

### Data description

Characteristics of our cohort within the development and validation datasets are presented in Table 1.

**Table 1:** Baseline characteristics of 352,608 mothers (development dataset) and 108,849 women (validation dataset).

	Development dataset (1997–2012)		Validation dataset (2013–2015)	
	Overall	PPD	Overall	PPD
*N* (%)	352,608 (100)	6402 (1.8)	108,849 (100)	2379 (2.2)
**CORE MODEL**				
Mothers’ age at birth, median (IQR)	28 (25, 31)	28 (25, 32)	28 (25, 31)	29 (26, 33)
Cohabitation (%)	395,051 (83.7)	5064 (79.1)	89,089 (81.8)	1886 (77.3)
Education (%)				
Mandatory	68,451 (19.4)	1723 (26.9)	16,460 (15.1)	532 (22.4)
Short	165,907 (47.1)	2855 (44.6)	41,383 (38.0)	912 (38.3)
Medium	16,340 (4.6)	221 (3.5)	5342 (4.9)	106 (4.5)
High	101,910 (28.9)	1603 (25.0)	45,664 (42.0)	829 (34.8)
Previous psychiatric history (%)				
None	282,076 (80.0)	1648 (25.7)	73,467 (67.5)	315 (13.2)
0-3 years before birth	19,848 (5.6)	1585 (24.8)	6519 (6.0)	576 (24.2)
3-10 years before birth	41,075 (11.6)	2788 (43.5)	20,446 (18.8)	1130 (47.5)
10+ years before birth	9609 (2.7)	381 (6.0)	8417 (7.7)	358 (15.0)
**ADDED PREDICTOR VARIABLES IN THE EXTENDED MODEL**				
Postpartum hemorrhage (%)	21,429 (6.1)	527 (8.2)	2,485 (2.3)	59 (2.5)
Gestational diabetes (%)	6531 (1.9)	219 (3.4)	3870 (3.6)	141 (5.9)
Gestational hypertension (%)	6835 (2.0)	171 (2.7)	3153 (2.9)	84 (3.5)
Preeclampsia (%)	15,055 (4.3)	363 (5.7)	5083 (4.7)	149 (6.3)
Eclampsia (%)	241 (0.1)	9 (0.1)	89 (0.1)	0 (0.0)
Previous stillbirths (%)	1881 (0.5)	37 (0.6)	474 (0.4)	14 (0.6)
Previous abortion (%)	82,709 (23.5)	1792 (28.0)	23,764 (21.8)	625 (26.3)
Acute C-section (%)	52,896 (15.0)	1262 (19.7)	16,934 (15.6)	457 (19.2)
Preterm birth (%)	22,226 (6.3)	609 (9.5)	5265 (4.8)	204 (8.6)
Hyperemesis gravidarum (%)	4899 (1.4)	200 (3.1)	2562 (2.4)	131 (5.5)
**ADDED PREDICTOR VARIABLES IN THE EXTENDED**+ **MODEL**				
Parents’ previous psychiatric history (%)	219,854 (62.4)	4967 (77.6)	82,260 (75.6)	2071 (87.1)
Charlson comorbidity index, within 10 years before birth (%)				
0	337,084 (95.6)	5944 (92.8)	102,589 (94.2)	2206 (92.7)
1	12,092 (3.4)	370 (5.8)	4779 (4.4)	130 (5.5)
2+	3432 (1.0)	88 (1.4)	1481 (1.4)	43 (1.8)

PPD is defined within 6 months after birth.

### Model development

In the univariate logistic regression models, all predictors were significantly associated with PPD except previous stillbirths, see Table 2. In particular, hyperemesis gravidarum (OR [95% CI] = 2.3 [2.0–2.7]) and gestational diabetes (OR [95% CI] = 1.9 [1.7–2.2] were associated with PPD, as well as previous psychiatric history among the mother and the mothers’ parents (OR [95% CI] = 2.1 [2.0–2.2]). Previous maternal psychiatric episodes increased PPD risk depending on how recent it was; with the ORs [95% CI] ranging from 7.0 [6.3–7.9] with psychiatric history more than 10 years to 14.8 [13.8-15.8] within 3 years prior to birth. A Wald test considering maternal age showed significant non-linearity in the log odds of PPD (χ^2^ = 98.4, df = 3, *p* < 0.0001). A transformation of maternal age using a third-degree polynomial provided the best fit, with a high value of *R*^2^ and a low AIC value in the univariate model and the lowest possible AIC when considering each of the three multivariate models. Mutual adjustment within the three multivariate models showed the same pattern as the univariate analyses, except for eclampsia and previous abortion, which now was not significant in any of the Extended models.

**Table 2:** Odds ratios (crude and mutually adjusted) between predictor variables and PPD within 6 months after birth.

	Odd Ratio, OR [95% CI]			
	Univariate	Multivariate (Core)	Multivariate (Extended)	Multivariate (Extended + )
Mothers age at birth				
Age (first order age)	0.54 (0.42-0.70)	0.74 (0.55-0.98)	0.72 (0.54-0.96)	0.74 (0.55-0.98)
Age^2^	1.02 (1.01-1.03)	1.01 (1.00-1.02)	1.01 (1.00-1.02)	1.01 (1.00-1.02)
Age^3^	1.00 (1.00-1.00)	1.00 (1.00-1.00)	1.00 (1.00-1.00)	1.00 (1.00-1.00)
Cohabitation	0.73 (0.69-0.78)	0.97 (0.91-1.04)	0.97 (0.91-1.03)	0.98 (0.92-1.04)
Education				
Mandatory	1.62 (1.51-1.73)	1.42 (1.30-1.54)	1.39 (1.28-1.51)	1.36 (1.25-1.48)
Short	1.10 (1.03-1.17)	1.17 (1.09-1.25)	1.16 (1.08-1.23)	1.15 (1.08-1.23)
Medium	0.86 (0.75-0.99)	0.89 (0.77-1.02)	0.88 (0.76-1.02)	0.88 (0.77-1.02)
High	1.00 (ref)	1.00 (ref)	1.00 (ref)	1.00 (ref)
Previous psychiatric history				
None	1.00 (ref)	1.00 (ref)	1.00 (ref)	1.00 (ref)
0-3 years before birth	14.77 (13.76-15.85)	14.28 (13.29-15.34)	14.09 (13.11-15.13)	13.49 (12.55-14.49)
3-10 years before birth	12.39 (11.65-13.18)	11.97 (11.25-12.74)	11.75 (11.04-12.51)	11.17 (10.48-11.89)
10+ years before birth	7.03 (6.27-7.87)	6.69 (5.97-7.50)	6.60 (5.88-7.40)	6.27 (5.59-7.03)
Postpartum hemorrhage (%)	1.40 (1.28-1.53)	―	1.39 (1.27-1.52)	1.38 (1.26-1.51)
Gestational diabetes (%)	1.91 (1.66-2.19)	―	1.32 (1.15-1.52)	1.32 (1.14-1.51)
Gestational hypertension (%)	1.40 (1.20-1.63)	―	1.21 (1.03-1.42)	1.20 (1.02-1.40)
Preeclampsia (%)	1.36 (1.22-1.51)	―	1.15 (1.03-1.29)	1.15 (1.03-1.29)
Eclampsia (%)	2.10 (1.08-4.09)	―	1.40 (0.70-2.81)	1.43 (0.71-2.87)
Previous stillbirths (%)	1.09 (0.78-1.51)	―	0.74 (0.53-1.03)	0.74 (0.53-1.03)
Previous abortion (%)	1.27 (1.21-1.35)	―	0.99 (0.93-1.05)	0.98 (0.93-1.04)
Acute C-section (%)	1.40 (1.32-1.49)	―	1.21 (1.13-1.29)	1.20 (1.13-1.29)
Preterm birth (%)	1.58 (1.45-1.72)	―	1.35 (1.23-1.47)	1.35 (1.23-1.47)
Hyperemesis gravidarum (%)	2.34 (2.03-2.71)	―	1.69 (1.46-1.96)	1.67 (1.44-1.93)
Charlson comorbidity score, within 10 years before birth		―	―	
0	1.00 (ref)	―	―	1.00 (ref)
1	1.76 (1.58-1.96)	―	―	1.11 (1.00-1.24)
2+	1.47 (1.19-1.81)	―	―	0.86 (0.69-1.07)
Parents previous psychiatric history (%)	2.12 (1.99-2.24)	―	―	1.44 (1.36-1.53)

Data from development dataset, 1997–2012, *N* = 352,608.

### Internal validation (reproducibility)

Performance of our three models was evaluated by calculating the predicted probability of PPD for each woman based on the estimated coefficients from the three developed models: Core, Extended, and Extended+. Within the development sample, the performance measured by the c-index ranged between 0.795-0.809, depending on how detailed the model was, Table 3. The average optimism of the performance within each model was small, resulting in an optimism-corrected c-index reducing the three c-estimates by a maximum of 0.001 (results not shown). This also implied that uniform shrinkage, using the optimism-corrected slope (Table 3), had limited influence on the models.

**Table 3:** Performance within internal and internal-external validation, including recalibrated intercept and slope of the three models.

	AUC (95%CI)	Calibration intercept	Calibration slope	CHCH test, p
*Internal, 1997–2012*				
Core	0.795 (0.790-0.802)	0.001^a^	1.000^a^	0.082
Ext	0.801 (0.796-0.807)	−0.007^a^	0.998^a^	0.060
Ext+	0.809 (0.804-0.815)	−0.005^a^	0.999^a^	0.003
*Internal-external, 2013–2015*				
Core	0.804 (0.796-0.812)	0.140	1.079	0.165^b^
Ext	0.808 (0.800-0.816)	0.107	1.068	0.144^b^
Ext+	0.812 (0.804-0.820)	0.091	1.074	0.126^b^

^a^The optimism-corrected calibration intercept and –slope, estimated using 200 bootstrapped samples. The slope is equivalent to the uniform shrinkage factor mentioned in the section Internal validation.

^b^The CHCH goodness of fit test is specified for the recalibrated models. The tests were rejected when considering the fit of the three developed models within the validation sample (*p* < 0.001, results not shown).

**Fig. 2:**
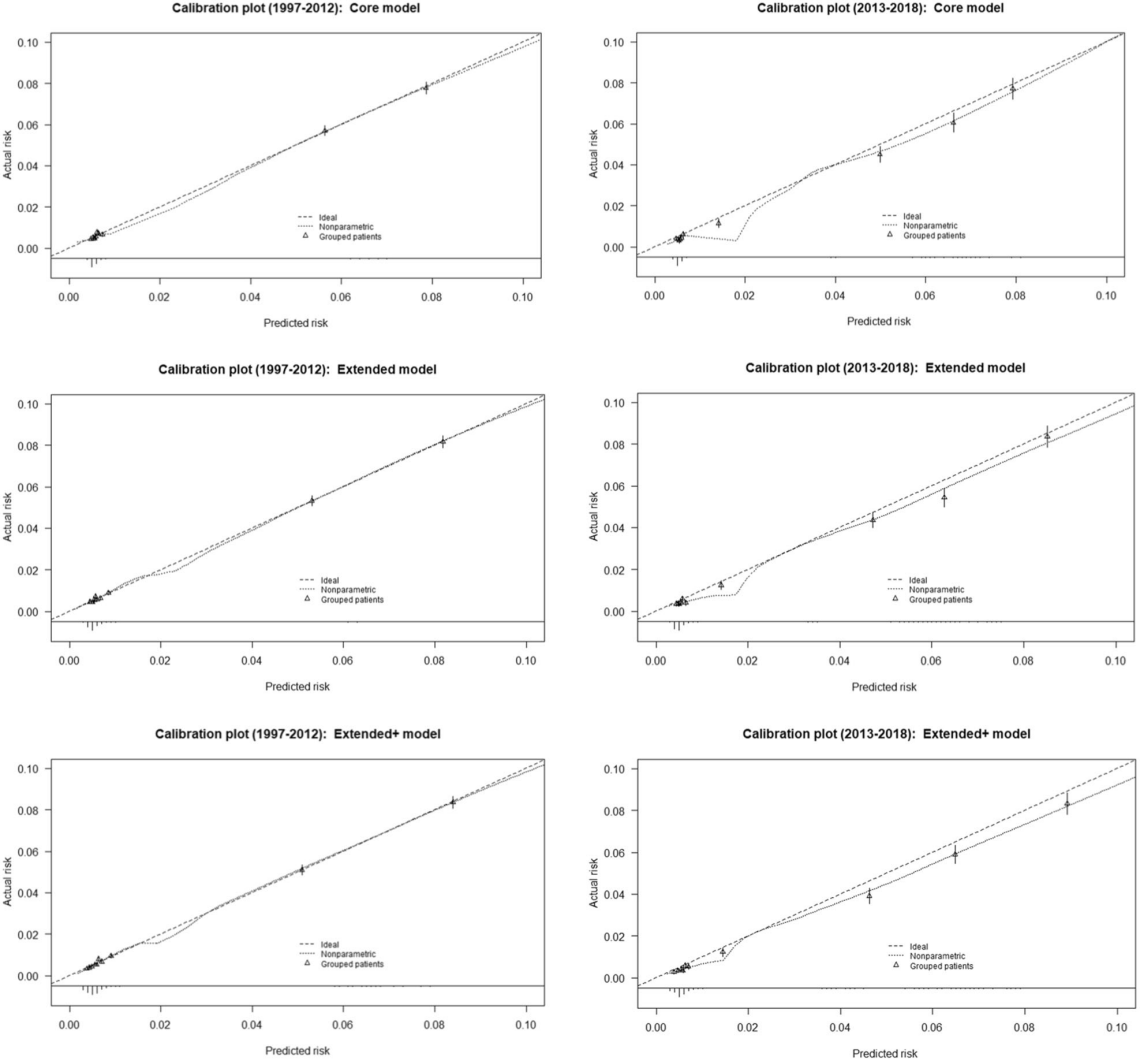
**Calibration plots.** Core, Extended and the Extended+ model for the development (years 1997–2012) and validation dataset (years 2013–2018).

The calibration plots showed low predicted probabilities primarily distributed between 0 and 0.01, Fig. 2. Within predicted probabilities of around 0.02, each of the three models overestimated the risk of PPD. However, the CHCH goodness of fit test was not rejected for the Core and Extended model [42], see Table 3.

### Internal-external validation (transportability)

The performance of the models within another dataset (the validation dataset), containing women with a first-time birth in 2013–2018 showed slightly higher discrimination for all three models. The c-index ranged from 0.804-0.812 depending on how detailed the model was (Table 3) but was not significantly different from the c-index within each model from the development sample (DeLong test, *p* = 0.07–0.53 respectively).

The calibration plots (Fig. 2) showed the three models overestimated the risk of PPD within the validation sample. This was confirmed by the-calibration-in-the-large intercept within all three models being below zero (−0.13 to −0.10, results not shown). The calibration slope was between 1.07–1.08 (Table 3), suggesting the model was slightly underfitting. The CHCH goodness of fit test was rejected for all three models.

Because all three models on average overestimated risk of PPD within the validation sample, we updated our models using recalibrating methods [50]. We adjusted the logistic regression coefficients corresponding to the recalibration model, logit(*Y*_val_ = *1*) *= a* + *b*_overall_ * LP_val_. These methods enhanced the calibration within all three models (Table 3) without changing the discrimination and the CHCH goodness of fit test was not rejected for any of the recalibrated models.

### Recommended final model

Discrimination increased depending on the number of variables included in the models, but was good for all three models, and remained good and not significantly different from the validation sample. In contrast, calibration was not optimal, as plots indicated all models overpredicted the probability of PPD, which worsened within the validation sample. To improve calibration, we adjusted our models using recalibration techniques, and all three recalibrated models could not be rejected according to the CHCH goodness of fit test. The calibration slope closest to 1 was seen in the Extended model, where the calibration-in-the-large also was the smallest (−0.101). We did not see substantial differences in performance between the three models, but based on our joint results recommend the recalibrated Extended model for future methods development due to its test performance. Coefficients in the Extended model can be found in Table 4. Moving forward, we expect this particular model will be relatively easy to use in a clinical setting.

**Table 4:** Coefficients in the Extended model.

	Development (1997–2012), α_dev_ and β_dev_	Validation (2013–2018), α_val_ and β_val_	Recalibrated model*
Intercept	−2.74	−4.22	−2.82
Mothers age at birth	−0.33	−0.30	−0.35
Age^2^	0.01	0.01	0.01
Age^3^	−0.00	−0.00	−0.00
Cohabitation	−0.03	0.12	−0.04
Education			
Mandatory	0.33	0.38	0.35
Short	0.14	0.16	0.15
Medium	−0.13	0.00	−0.13
High	Ref	Ref	Ref
Previous psychiatric history			
None	Ref	Ref	Ref
Within 3 years before birth	2.65	3.12	2.83
3–10 years before birth	2.46	2.55	2.63
10+ years before birth	1.89	2.14	2.02
Postpartum hemorrhage (%)	0.33	0.02	0.35
Gestational diabetes (%)	0.28	0.23	0.30
Gestational hypertension (%)	0.19	0.05	0.20
Preeclampsia (%)	0.14	0.16	0.15
Eclampsia (%)	0.34	−7.33	0.36
Previous stillbirths (%)	−0.30	−0.06	−0.32
Previous abortion (%)	−0.01	−0.12	−0.01
Acute c-section (%)	0.19	0.03	0.20
Preterm birth (%)	0.30	0.49	0.32
Hyperemesis gravidarum (%)	0.53	0.63	0.56

*To calculate the coefficients in the recalibrated model, the estimates α_new_ and β_overall_ has to be added and multiplied, respectively, to the original model (here the Extended model).

**Fig. 3:**
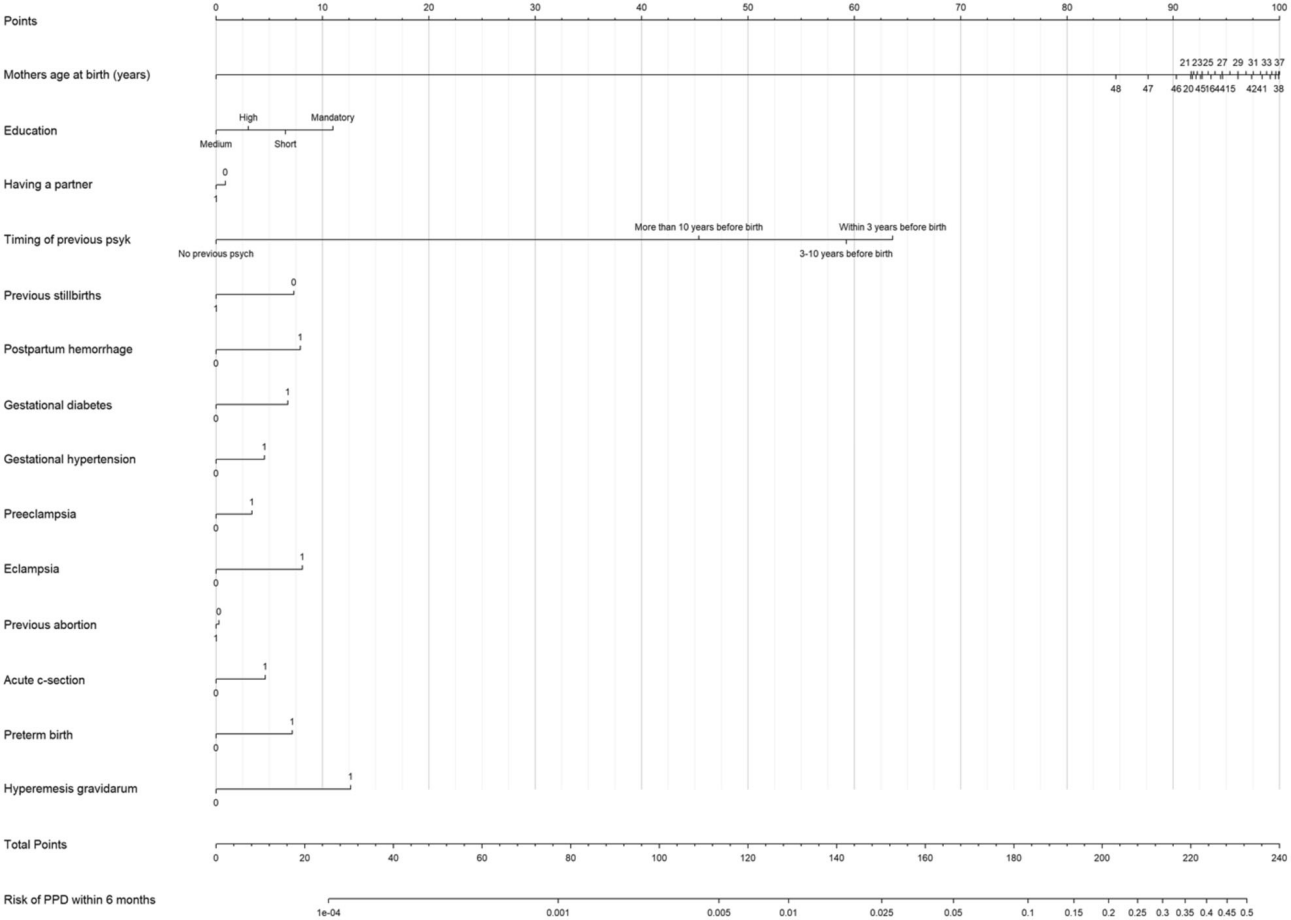
Nomogram.

The final model (recalibrated version of the Extended model) is presented in a nomogram (Fig. 3), illustrating each predictor assigned certain points (first line in the figure), and can be used the following way: A woman’s probability of PPD can be calculated by summing up all individual points assigned to the woman’s covariates, and the total number of points is then translated into the probability of PPD, illustrated by the lowest two lines in the figure. As demonstrated in Fig. 3, predictors affecting the probability of PPD the most were previous psychiatric history closest to childbirth, maternal age around mid/late thirties, a low education, and hyperemesis gravidarum, corresponding to points around 100, 64, 11, and 13, respectively.

Finally, a sensitivity of 0.87, a specificity of 0.69, and a positive predictive value (PPV) of 0.06 were observed within the validation sample. This was calculated with the predicted probabilities from the recalibrated Extended model, applying a threshold of 0.025 defined by maximizing the sum of sensitivity and specificity (Table 5a). Within the combined dataset, consisting of 461,457 women, the sensitivity decreased, and the specificity increased to 0.78 with the same optimal threshold and a slightly higher PPV (Table 5b). Considering increasing threshold values applied in Table 5b, we as expected observed the sensitivity decreased while the specificity and PPV increased.

**Table 5:** Sensitivity and specificity in the recalibrated Extended model.

(a) Confusion matrix, threshold = 0.025		
Threshold = 0.025	PPD	No PPD
Predicted PPD: yes	6813	98,754
Predicted PPD: no	1968	353,922

(b) Sensitivity and specificity for different threshold values, within the combined dataset consisting of 461,457 women			
Threshold	Sens	Spec	PPV
0.005	0.844	0.597	0.039
0.025^a^	0.776	0.782	0.065
0.05	0.657	0.837	0.072
0.1	0.059	0.991	0.114
0.15	0.005	0.999	0.144

^a^Maximizes the sum of sensitivity and specificity.

## Discussion

For the present study, we developed three PPD prediction models (Core, Extended, and Extended+) in a large population-based cohort. Discrimination and calibration were best within the Extended and Extended+ models. As we found a negligible difference in performance between the Extended and Extended+ model, we recommend the recalibrated Extended model for further development in future work. Moving forward, we also speculate that this model can be implemented in the clinical setting whenever feasible.

Our models have been developed, acknowledging how this work could in the future guide clinicians in their decision making about additional testing, informing patients about their individualized risk with the use of a risk-calculator (https://ncrr-au.shinyapps.io/PPDRiskCalc/), but also support considerations about time-sensitive and cost-effective treatment/intervention [51]. In the future, prediction models hopefully will augment clinical decisions as suggested by Steyerberg et al. [52], raise clinician awareness of PPD, guide interventions, increase screening and referral rates [18], and through this, prevent serious psychiatric episodes. However, we strongly emphasize multiple steps are needed before our model or similar work can be implemented in real-world clinical routines. We note that this is a challenge not only related to PPD prediction, as Meehan et al. recently found only one out of 308 published prediction models within psychiatry was formally evaluated and assessed for usefulness in clinical care [53]. We propose that moving forward any efforts toward clinical implementation could include engagement with stakeholders, including patients, clinicians, and politicians, to evaluate how to maximize the translational potential of our model as well as models from other groups.

### Prediction of PPD risk

Of the existing published papers on PPD risk prediction, several have applied machine learning approaches [17–20]. Among others, these studies considered predictor variables related to previous mental health, socioeconomic status, as well as obstetric and childbirth-related variables, and found past history of depression and anxiety as some of the most important predictor variables, as well as stress in pregnancy. Aggregate measures of model performance varied from 81 to 93 (AUCs) compared to AUCs of about 80 in the present study (Table 3), but direct comparisons are challenging as studies used different statistical methods, defined PPD at different time points and through different PPD measures, including self-reported symptoms not included in population registers, psychiatric admissions, and prescription drug use (antidepressants).

### Methodological considerations, limitations, and next steps

To our knowledge, our study included the largest sample to date with 6402 PPD cases (development sample) and 2379 PPD cases (validation sample), and our cohort represents a national population of primiparous women diminishing potential bias due to attrition and low response rates. We suggest our Extended model will be preferable moving forward, among others, based on an acceptable AUC, discrimination, and calibration. However, our model is yet to be validated in an external dataset and this would be the next development step in our work. We also consider adding additional predictor variables to the model and test how this affects model performance, while at the same time remembering predicting rare outcomes and new PPD episodes is challenging [20, 54]. PPD prevalence in our current sample is only around 2%, reflecting the *diagnosis/treatment* prevalence but not the *illness* prevalence. Hence, another next step, after further validation, would be to evaluate whether the model can be used for a different outcome, e.g., PPD defined by symptoms assessed using the Edinburg Postnatal Depression Scale (EPDS) [55]. Limitations of the study must be considered, including the generalizability of our findings including considering to which extent our models reflect the Danish health care system and treatment standards for more severe PPD episodes, as well as acknowledging our list of PPD predictors is far from exhaustive. We additionally speculate that for subgroups of particularly vulnerable mothers, aspects related to socioeconomic conditions may trump our included variables, and issues related to e.g. history of trauma or immigration status will be highly relevant to consider moving forward. However, this type of information was unfortunately not available in our dataset and hence could not be included.

As pointed out by Fusar-Poli et al., more models are developed than are used in clinical settings, likely because many are too complex [51]. We prioritized including predictor variables in our work that are clinically applicable, easy to identify, and rely on information that should be readily available at time of delivery. We also prioritized presenting results for both discrimination, calibration, and validation, all aspects being equally important when developing prediction models. However, we acknowledge that prognostic models with increasing complexity could be relevant and preferable in cases where prediction ability also is improved. Such an expansion of the model could include self-reported measures (e.g., maternal resilience), as well as genetic vulnerability measured as polygenic scores or biomarkers measuring hormonal sensitivity, which all have been linked to PPD risk [18, 20, 56–58]. Moreover, we acknowledge that our final recommended model will have a substantial proportion of false positive tests, but importantly also capture 76% of the women who will end up developing PPD. In summary, we here argue that the field of perinatal psychiatry may not need more PPD prediction models, but progress can be ensured through existing models being further validated, expanded, and strengthened in relation to performance and reproducibility, calling for standardized data collection and extended collaborations.

In conclusion, we developed three prediction models for PPD (Core, Extended, and Extended+), and validated and recalibrated them accordingly. Our recalibrated Extended model with 14 variables achieved the highest performance, with satisfying calibration and discrimination. Previous psychiatric history, maternal age, low education, and hyperemesis gravidarum were the most important identified predictors in our final PPD prediction model in primiparous women.

The developed risk calculator is available online but is not at present ready for direct implementation in clinical care before additional validation has been performed. A future developed and validated PPD prediction model could ideally assist and add to prevention efforts, as recently recommended by the US Preventive Services Task Force [12]. A more specific focus on *who* will benefit from preventive PPD interventions is, however, to our knowledge, not included in any of the existing published PPD prediction models but will be an evident next step for the research field. This will be particularly relevant as part of a discussion on possible adjustments for how and when systematic screening can supplement the prediction of PPD, while also considering both potential consequences from false positive and false negative test results applied using different threshold values and which protective effects can reduce PPD risk.

## Supplementary information

Supplementary table
