# Supplementary material for: Postpartum depression: a developed and validated model predicting individual risk in new mothers
Source: Transl Psychiatry. 2022 Sep 30;12:419. doi: 10.1038/s41398-022-02190-8 (PMC9525696; doi:10.1038/s41398-022-02190-8)
Supplement: Supplementary file 1 — Supplementary table [file 41398_2022_2190_MOESM1_ESM.docx]

Supplementary table 1: Identification and definition of included predictor variables.

|  | **Variable type** | **Timing** | **Core Model** | **Ext Model** | **Ext+ Model** | **Coding** |
| --- | --- | --- | --- | --- | --- | --- |
| Mothers age | Continous | At birth | X | X | X | Mothers age in years |
| Cohabitation | Binary;   - Married, cohabiting - single, divorced, widowed | January 1, the year (if missing, the year before) where the child is born | X | X | X |  |
| Education | Categorical;   - Mandatory (≤9 years) - Short (secondary school / vocational education, aprox ≤10-12 years) - Medium (short further education) - High (medium/long-term higher education, aprox >12 years) | October 1, the year (if missing, two years) before date of birth | X | X | X |  |
| Previous psychiatric history | Categorical;   - None - 0-3 years before birth - 3-10 years before birth - 10+ years before birth | Before 6 months prior to conception | X | X | X | ICD-8 (LPR/PCR): 290-315  ICD-10 (LPR/PCR): F00–F99  NPR: N03-N07 |
| Postpartum hemorrhage | Binary; yes/no | At birth |  | X | X | ICD-10 (LPR): O72 |
| Gestational diabetes | Binary; yes/no | During pregnancy |  | X | X | ICD-10 (LPR): O24 |
| Gestational hypertension | Binary; yes/no | During pregnancy |  | X | X |  |
| Preeclampsia | Binary; yes/no | During pregnancy |  | X | X | ICD-10 (LPR): O14 |
| Eclampsia | Binary; yes/no | During pregnancy |  | X | X | ICD-10 (LPR): O15 |
| Previous stillbirths* | Binary; yes/no | Before pregnancy |  | X | X | MBR and CRS |
| Previous abortion | Binary; yes/no | Before pregnancy |  | X | X | ICD-8 (LPR): 634.0, 634.61, 640-642, 643.8, 643.9, 645.1, 779  ICD-10 (LPR): O02.1, O03-O07 |
| Acute c-section | Binary; yes/no | At birth |  | X | X | ICD-10 (LPR): O82.1, O84.3, and  MFR: b_sectiou=1 and/or KMCA10A/E |
| Preterm birth | Binary; yes/no | At birth |  | X | X | ICD-10 (LPR): O60, and  MFR: duration of pregnancy period within 148-258 days. |
| Hyperemesis gravidarum | Binary; yes/no | During pregnancy |  | X |  | ICD-10 (LPR): O21 |
| Parents previous psychiatric history | Binary; yes/no | Before date of birth |  |  | X | ICD-8 (LPR/PCR): 290-315  ICD-10 (LPR/PCR): F00–F99  NPR: N03-N07 |
| Charlson Comorbidity Index | Number of diseases included in Charlson’s comorbidity index, categorized as;  0, 1, 2+ | Within 10 years before date of birth |  |  | X | **Myocardial Infarction** ICD-8 (LPR): 410  ICD-10 (LPR): I21-I23  **Congestive Heart Failure** ICD-8 (LPR): 427.09-427.11, 427.19, 428.99, 782.49  ICD-10 (LPR): I50, I11.0, I13.0, I13.2  **Peripheral Vascular Disease** ICD-8 (LPR): 440-445  ICD-10 (LPR): I70-I74, I77  **Cerebrovascular Disease**  ICD-8 (LPR): 430-438  ICD-10 (LPR): I60-I69, G45-G46  **Dementia**  ICD-8 (LPR): 290.09-290.19, 293.09  ICD-10 (LPR): F00-F03, F05.1, G30  **Chronic Pulmonary Disease**  ICD-8 (LPR): 490-493, 515-518  ICD-10 (LPR): J40-J47, J60-J67, J68.4, J70.1, J70.3, J84.1, J92.0, J96.1, J98.2, J98.3  **Connective Tissue Disease**  ICD-8 (LPR): 712, 716, 734, 446, 135.99  ICD-10 (LPR): M05-M06, M08-M09, M30-M36, D86  **Ulcer Disease**  ICD-8 (LPR): 530.91, 530.98, 531-534  ICD-10 (LPR): K22.1, K25-K28  **Mild Liver Disease**  ICD-8 (LPR): 571, 573.01, 573.04  ICD-10 (LPR): B18, K70.0-K70.3, K70.9, K71, K73-K74, K76.0  **Diabetes I and II**  ICD-8 (LPR): 249.00, 249.06, 249.07, 249.09, 250.00, 250.06, 250.07, 250.09  ICD-10 (LPR): E10.0, E10.1, E10.9-E11.1, E11.9  **Hemiplegia**  ICD-8 (LPR): 344  ICD-10 (LPR): G81-G82  **Moderate to Severe Renal Disease**  ICD-8 (LPR): 403-404, 580-584, 590.09, 593.19, 753.10-753.19, 792  ICD-10 (LPR): I12-I13, N00-N05, N07, N11, N14, N17-N19, Q61  **Diabetes with End Organ**  ICD-8 (LPR): 249.01-249.05, 249.08, 250.01-250.05, 250.08  ICD-10 (LPR): E10.2-E10.8, E11.2-E11.8  **Any Tumor**  ICD-8 (LPR): 140-194  ICD-10 (LPR): C00-C75  **Leukemia**  ICD-8 (LPR): 204-207  ICD-10 (LPR): C91-C95  **Lymphoma**  ICD-8 (LPR): 200-203, 275.59  ICD-10 (LPR): C81-C85, C88, C90, C96  **Moderate to Severe Liver Disease**  ICD-8 (LPR): 070.00, 070.02, 070.04, 070.06, 070.08, 573.00, 456.00-456.09  ICD-10 (LPR): B15.0, B16.0, B16.2, B19.0, K70.4, K72, K76.6, I85  **Metastatic Solid Tumor**  ICD-8 (LPR): 195-199  ICD-10 (LPR): C76-C80  **AIDS**  ICD-8 (LPR): 079.83  ICD-10 (LPR): B21-B24 |

*During 2016-2018 there was missing data on stillbirths and date of conception (date of conception defined as 280 days before birth, if missing).
